# Long-Term Vineyard Cultivation Reshapes Soil Organic Phosphorus Speciation, Phosphatase Activities, and *phoC*- and *phoD*-Harboring Bacterial Communities

**DOI:** 10.3390/microorganisms14091991

**Published:** 2026-09-09

**Authors:** Shuo Fang, Lei Yan, Xue Wang, Zhubing Shao, Yuxuan Shao, Ye Liu, Chunyan Yu, Xiaotong Guo, Guohui Wu, Yu Zhang

**Affiliations:** 1 College of Horticulture, Ludong University, Yantai 264025, China; 19553103698@163.com (S.F.); 15864008385@163.com (L.Y.); wxyzxy1014@163.com (X.W.); 19560868160@163.com (Z.S.); 17854659533@163.com (Y.S.); 18562380689@163.com (Y.L.); yuchunyan@ldu.edu.cn (C.Y.); guoxtina@ldu.edu.cn (X.G.); 2 Yantai Key Laboratory of Crop Molecular Breeding for High-Yield and Stress-Resistant Crops and Efficient Cultivation, Yantai 264025, China; 3 Institute of Forestry and Pomology, Beijing Academy of Agriculture and Forestry Sciences, Beijing 100093, China

**Keywords:** P-mineralizing bacteria, *phoC* and *phoD* genes, soil organic P forms, solution ^31^P NMR, phosphatase activity, vineyard

## Abstract

Organic phosphorus (OP) transformation in perennial vineyards is strongly influenced by long-term cultivation, fertilization, and associated changes in soil properties. However, the relationships among soil OP speciation, phosphatase activities, and *phoC*- and *phoD*-harboring bacterial communities remain poorly understood. In this study, soils from 20-year-old vineyards (Y20) and adjacent uncultivated reference sites (CK) in Penglai, China, were compared. Soil available P was significantly higher in Y20 than in CK, increasing from 15.83 to 40.50 mg kg^−1^, whereas total P, OP, and microbial biomass P did not differ significantly between treatments. The contents of soil orthophosphate and corrected monoesters (cMonoesters) were in the range of 15.34–97.45 and 6.90–24.49 mg kg^−1^, respectively, and were significantly higher in Y20 than in CK. Among the identified monoesters, the mean concentrations of *myo*-inositol hexakisphosphate (*myo*-IHP), *scyllo*-IHP, and choline phosphate in Y20 were 4.12, 0.69 and 0.69 mg kg^−1^, respectively, significantly higher than those observed in CK. The Y20 soils also exhibited 17.94% higher acid phosphomonoesterase (ACP) activity and 28.05% lower alkaline phosphomonoesterase (ALP) activity than the CK soils. Furthermore, the α-diversity of *phoC*-harboring bacteria was lower in Y20, whereas the composition of *phoD*-harboring communities differed significantly between the two land-use types. Correlation analyses showed that soil OP forms correlated positively with ACP activity and *phoD* community composition, but negatively with ALP activity. Overall, the differences between Y20 and CK suggest that long-term vineyard cultivation was associated with increased P availability through redistribution among soil P forms and with the accumulation of relatively stable cMonoesters. These patterns were closely related to phosphomonoesterase activities and the composition of *phoD*-harboring bacterial communities. These findings highlight the potential interactions among P speciation, phosphatase activities, and functional microbial communities in vineyard soils and provide hypotheses for further investigation of P transformation processes.

## 1. Introduction

As a key plant macronutrient, phosphorus (P) supports crop growth and yield formation by participating in fundamental processes such as energy metabolism, photosynthesis, root development, and nutrient uptake. These functions are especially important for grapevine growth and productivity. Nevertheless, the efficient management of P remains difficult in intensively managed perennial systems, where long-term cultivation and repeated fertilizer inputs may alter soil P availability and increase the risk of P accumulation and loss. Vineyards often receive repeated P fertilizer applications to sustain long-term productivity [1,2]. However, fertilizer-derived P may be rapidly sorbed by soil minerals, precipitated into less soluble forms, or temporarily incorporated into microbial biomass, leaving only a small fraction immediately available to plants [3,4,5]. Continued P inputs can therefore result in the accumulation of legacy P, which may later be remobilized and transported to adjacent water bodies, increasing the risk of eutrophication [6,7]. Moreover, long-term agricultural management affects not only total soil P (TP) but also its distribution among chemical forms that differ in stability, mobility, and biological accessibility [8,9]. Understanding how these P forms accumulate and transform is therefore critical for optimizing fertilizer use and minimizing P losses from agricultural soils.

Soil organic P (OP) constitutes a substantial and chemically diverse fraction of total soil P and can contribute to plant-available P (AP) through mineralization [10,11]. The persistence and bioavailability of OP depend strongly on its molecular composition and interactions with soil minerals [12]. Phosphomonoesters, particularly compounds containing multiple phosphate groups, generally have a high affinity for reactive Al and Fe oxides and mineral edge sites, whereas phosphodiesters are less strongly retained and are often more susceptible to biological turnover [13,14]. The characterization of soil OP has traditionally been limited by the poor extraction, separation, and detection of chemically complex compounds [15]. The combined use of NaOH-EDTA extraction and solution ^31^P nuclear magnetic resonance (NMR) spectroscopy has substantially improved the molecular characterization of soil P by enabling different P groups to be identified according to their chemical shifts [16,17,18]. Across a wide range of soils, extractable OP is commonly dominated by phosphomonoesters and phosphodiesters [13,19,20,21,22]. Nevertheless, the effects of long-term grapevine cultivation on the molecular composition and transformation of soil OP remain poorly understood.

The conversion of OP into bioavailable orthophosphate is mediated primarily by extracellular phosphatases, including acid phosphomonoesterase (ACP), alkaline phosphomonoesterase (ALP), and phosphodiesterase (PDE) [13,22,23,24]. The functional genes *phoC* and *phoD*, which are associated mainly with bacterial ACP and ALP production, respectively, are widely used as molecular markers for assessing the diversity, composition, and potential functions of P-mineralizing bacterial communities [25,26,27]. Previous studies have shown that long-term cultivation and fertilization can alter soil P pools, phosphatase activities, and these functional microbial communities. For example, OP concentrations were higher in pomelo orchards cultivated for more than 10 years than in background soils and younger orchards [9]. Long-term citrus cultivation increased soil P availability but intensified acidification and reduced the diversity of *phoC*- and *phoD*-harboring bacteria, *phoD* abundance, and ALP activity [8]. Similarly, 30 years of citrus cultivation was associated with inorganic P accumulation, lower soil pH and organic matter, reduced *phoD*-harboring bacterial diversity and abundance, and altered P-mineralizing bacterial community composition [28]. Citrus cultivation over 5–15 years also modified soil pH and organic carbon, which subsequently affected the structure, diversity, and co-occurrence network of *phoD*-harboring bacteria, as well as ALP activity and AP [29].

Fertilization can influence OP mineralization through direct changes in nutrient availability and indirect effects on microbial communities. Fertilization affects the abundance and composition of *phoD*-harboring bacteria in different ecosystems [30,31,32,33]. Long-term P addition can regulate ALP activity by altering *phoD* abundance, bacterial diversity, and community composition [34]. However, the direction and magnitude of these effects are not always consistent. High P application rates reduced ACP activity and *phoC* abundance in an alkaline sandy loam [35], whereas chemical nitrogen (N) and P inputs have been reported to enhance ACP and ALP activities in acidic soils, partly by modifying *phoC*- and *phoD*-harboring bacterial communities and increasing microbial biomass P (MBP) [25]. In acidic soils, maize rhizosphere effects were stronger than those of P fertilization in shaping phosphatase activities and *phoC*-harboring bacterial communities [36]. Collectively, these findings indicate that the effects of cultivation and fertilization on OP mineralization depend on their interactions with soil properties, crop-microbe relationships, fertilizer regime, and the duration of nutrient enrichment.

Soil pH is a particularly important regulator of phosphatase-mediated P cycling because it influences enzyme conformation, substrate affinity, inhibition, and catalytic efficiency [37]. Soil ACP is generally more active in acidic soils, whereas ALP is typically favored under neutral to alkaline conditions [38,39]. However, soil acidification can produce contrasting responses. Enhanced ACP activity has been observed in some acidified agricultural soils [40], whereas other studies have reported reductions in ACP and other P-cycling enzymes with declining pH [41,42]. These inconsistencies suggest that the effects of acidification depend on the interactions among pH, substrate availability, nutrient status, microbial community composition, and enzyme origin. In addition, inorganic P availability commonly exerts negative feedback on ALP production, as increased P availability may reduce microbial investment in P-acquiring enzymes [8,34]. Although both plants and microorganisms produce phosphatases, ACP is derived from plant roots and microorganisms, whereas ALP is predominantly associated with microbial activity [39]. Root-derived ACP may thus partially compensate for changes in microbial *phoC*-mediated P-mineralization potential under environmental stress [26,43].

The ecological responses of *phoC*- and *phoD*-harboring bacteria may also differ because these groups occupy distinct ecological niches and adopt contrasting resource-use strategies [25]. Many *phoC*-harboring taxa exhibit oligotrophic characteristics and may persist under nutrient-limited conditions, potentially making them relatively resistant to environmental fluctuations [44,45]. By contrast, *phoD*-harboring bacterial communities appear to be more sensitive to local soil conditions [25]. Soil pH, AP, C:P stoichiometry, and the availability of OP substrates have been identified as important factors controlling their diversity and composition [10,27,43,46,47]. Despite these advances, it remains unclear how *phoC*- and *phoD*-harboring bacteria are related to specific OP compounds under long-term agricultural disturbance. This knowledge gap is particularly evident in vineyards, where continuous litter inputs, extensive root systems, and recurrent fertilizer applications may jointly reshape soil P speciation, enzyme activity, and microbial habitats.

To address this gap, we conducted a space-for-time comparison between soils from 20-year-old vineyards and adjacent uncultivated sites in Penglai, China. By integrating solution ^31^P NMR spectroscopy, phosphatase assays, and high-throughput sequencing of the *phoC* and *phoD* genes, we aimed to: (1) evaluate differences in soil P pools, individual P compounds, phosphatase activities, and P-mineralizing bacterial communities between 20-year-old vineyard soils and adjacent uncultivated soils; and (2) identify the potential relationships among soil P speciation, phosphatase activity, and functional bacterial assemblages. We hypothesized that: (1) soils under long-term vineyard management would exhibit different P speciation patterns, including a relative accumulation of stable phosphomonoester compounds, compared with adjacent uncultivated soils; and (2) the environmental conditions associated with long-term vineyard management would be associated with contrasting patterns in *phoC*- and *phoD*-harboring bacteria, leading to functional communities shifts associated with changes in phosphatase activities and the accumulation or depletion of specific OP compounds. By integrating molecularly resolved P composition, phosphatase activity, and functional bacterial community data, this study provides new insights into the potential factors associated with soil P dynamics in perennial vineyard systems.

## 2. Materials and Methods

### 2.1. Site Description and Soil Sampling

**Figure A1:**
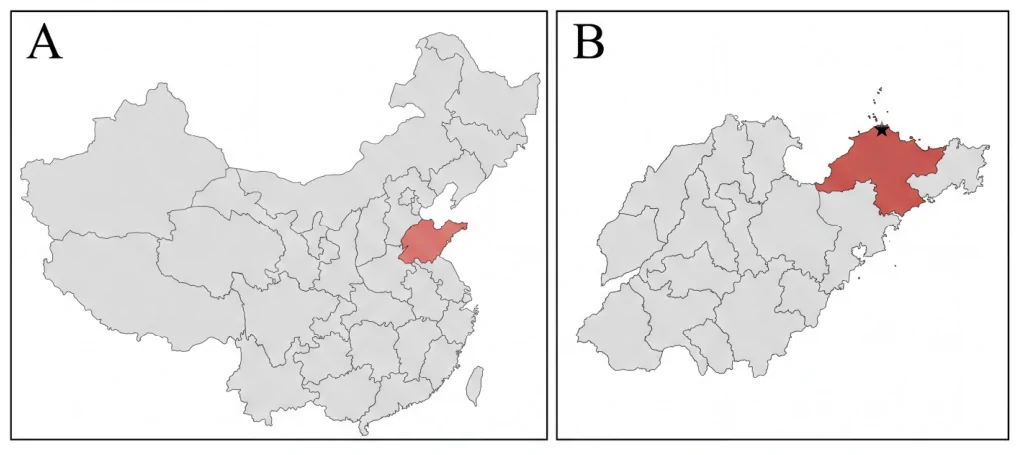
Location of the study area. (**A**) Location of Shandong Province in China; (**B**) detailed view of Shandong Province, with the black star indicating the sampling site. Reproduced from Wu et al. [88].

A space-for-time comparative study was established in Penglai District, Yantai, Shandong Province, China (37°45′ N, 120°50′ E; Figure A1). This region has a warm-temperate continental monsoon climate, with a mean annual temperature of 12.9 °C and annual precipitation of approximately 661 mm. The soil is classified as brown soil (Udic Luvisols, WRB) and has a sandy loam texture. In September 2022, soils were sampled from four vineyards with a 20-year cultivation history (Y20) and four nearby uncultivated reference sites (CK). The CK sites were uncultivated wastelands located near the vineyards and had received neither crop cultivation nor fertilizer inputs. At the time of sampling, they were covered by some natural herbaceous vegetation. The Y20 and CK sites were selected from the same general soil and landscape unit and had similar topography, elevation and slope, to reduce potential differences in parent material and other environmental conditions. However, detailed information on the historical vegetation, parent-material composition, and pre-establishment soil properties of the sites was not available. The vineyard received water-soluble fertilizer via drip fertigation three to four times per year, at an application rate of 75 kg ha^−1^ each time. Annual postharvest fertilization included 750 kg ha^−1^ of compound fertilizer. Moreover, fertilizers containing secondary nutrients and micronutrients—including Ca, Mg, Mn, and Zn—were applied at a combined rate of 375–600 kg ha^−1^ yr^−1^ [1]. Additional information on current vineyard fertilization practices was collected where available. However, historical records of fertilizer formulations, application rates, and annual P or P_2_O_5_ inputs were not available, and therefore long-term P inputs could not be quantified. Similarly, long-term records on organic amendments, tillage practices, and cover-crop management were unavailable. At each site, five randomly distributed cores (0–20 cm depth; 5 cm diameter) were collected and combined into one composite sample. After homogenization, soils were sieved through a 2 mm mesh. Fresh subsamples were stored at −80 °C for DNA extraction and at 4 °C for phosphatase activity and microbial biomass P (MBP) analyses. The remaining soil was air-dried for routine chemical characterization and solution ^31^P NMR analysis.

### 2.2. Soil Properties Analysis

Soil total P (TP) was quantified after HClO_4_ digestion, whereas organic P (OP) was determined by means of the ignition procedure [48]. Soil MBP was measured using CHCl_3_ fumigation-extraction [49], and available P (AP) was recovered with 0.5 M NaHCO_3_ [50]. Orthophosphate in all extracts was determined colorimetrically using the molybdate method at 880 nm [51]. Soil pH and SOC data were obtained from Fang et al. [1]. Soil pH was measured using a digital pH meter at a soil-to-water ratio of 1:2.5 *w*/*v*. The SOC content was determined using the K_2_Cr_2_O_7_ oxidation method [52]. The C:P ratio was calculated as the mass ratio of SOC to TP. The soil properties used in the present study are presented in Table 1.

**Table 1:** Soil properties used in the present study under the two treatments.

Soil Property	CK	Y20	Test Statistic	*p* Value	Data Source
pH	7.55 ± 0.46	6.57 ± 0.12	4.156	0.006	Fang et al. [1]
SOC (g kg^−1^)	10.2 ± 1.8	11.54 ± 1.35	−1.194	0.277	Fang et al. [1]
TP (mg kg^−1^)	614.89 ± 163.7	605.87 ± 60.6	0.103	0.921	Figure 1
OP (mg kg^−1^)	116.68 ± 46.77	106.67 ± 4.81	0.425	0.699	Figure 1
MBP (mg kg^−1^)	8.5 ± 1.12	9.38 ± 1.11	−1.119	0.306	Figure 1
AP (mg kg^−1^)	15.83 ± 15.08	40.5 ± 12.76	−2.498	0.047	Figure 1
C:P	17.75 ± 6.8	19.27 ± 3.67	−0.393	0.708	present analysis

**Figure 1:**
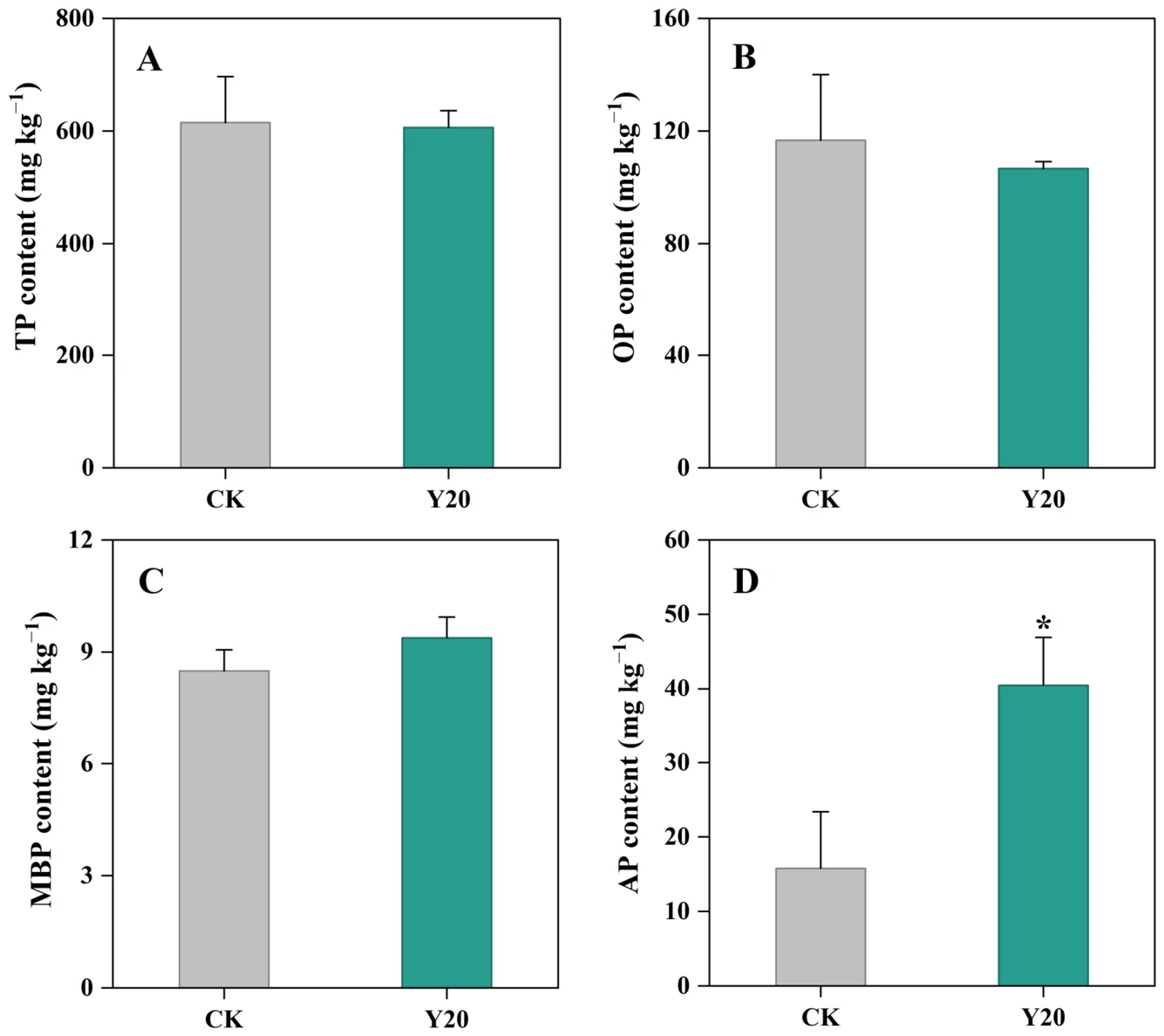
Changes in soil P pools under CK and Y20. Values are means and error bars represent standard errors (*n* = 4). (**A**) TP: total P; (**B**) OP: organic P; (**C**) MBP: microbial biomass P; (**D**) AP: available P. CK: uncultivated soils; Y20: 20-year cultivated vineyard. * indicates significant differences between CK and Y20 (*p* < 0.05).

Values are presented as mean ± SD (*n* = 4). Soil pH and SOC data were obtained from the experimental dataset previously reported by Fang et al. [1] and are included here to document the soil variables used in the present study. TP, OP, MBP, and AP are also presented in Figure 1. The C:P ratio was calculated from the SOC and TP concentrations. SOC: soil organic carbon; TP: total P; OP: organic P; MBP: microbial biomass P; AP: available P. CK: uncultivated soils; Y20: 20-year cultivated vineyard.

### 2.3. Determination of Soil P Species by Solution ^31^P NMR

For solution ^31^P NMR analysis, 3.0 g of soil from each replicate plot was shaken with 30 mL of 0.25 M NaOH-0.05 M Na_2_EDTA at 20 °C for 16 h and then centrifuged [53]. A 1 mL portion of each extract was analyzed for P, Fe, Mn, Al, and Ca using inductively coupled plasma-optical emission spectroscopy (ICP-OES; Optima 5300DV, PerkinElmer, Shelton, CT, USA). The residual extract was stored at −40 °C before freeze-drying. Each freeze-dried extract was redissolved using 0.65 mL each of D_2_O, deionized water, and NaOH-EDTA solution, together with 0.40 mL of 10 M NaOH, and transferred to a 10 mm NMR tube. Spectra were acquired without proton decoupling on a Bruker Avance 500 MHz spectrometer using a 90° pulse, an acquisition time of 0.68 s, a relaxation delay of 5 s, and 2800–6000 scans. The relaxation delay was selected according to the extract P/(Fe + Mn) ratio [16,54]. Individual P compounds were identified from published chemical-shift data and confirmed by adding selected standards, including *myo*-inositol hexakisphosphate (*myo*-IHP), α-glycerophosphate (α-Glyc), β-glycerophosphate (β-Glyc), and choline phosphate (Pchol), to representative samples [23,55,56,57,58]. The assignments and chemical shifts of the detected P species are provided in Table A1. Peak areas were calculated by integrating spectra processed with 2- and 7-Hz line broadening in NMR Utility Transform Software 2000 (NUTS; Acorn NMR, Livermore, CA, USA).

### 2.4. Phosphatase Activity Analysis

Soil ACP, ALP, and PDE activities were determined following Tabatabai [24]. Soil ACP and ALP were measured with p-nitrophenyl phosphate as the substrate in modified universal buffers adjusted to pH 6.5 and 11.0, respectively. Soil PDE activity was measured at pH 8.0 using bis-p-nitrophenyl phosphate. All enzyme activities were expressed as mg p-nitrophenol kg^−1^ soil h^−1^.

### 2.5. Extraction of Soil DNA and High-Throughput Sequencing

Total DNA was isolated from 0.50 g of each soil sample using the FastDNA^®^ Spin Kit for Soil (MP Biomedicals, Solon, OH, USA). DNA integrity and concentration were evaluated by 1% agarose gel electrophoresis and a NanoDrop 2000 spectrophotometer (Thermo Fisher Scientific, Wilmington, DE, USA), respectively. Bacterial *phoC* fragments were amplified with *phoC*-A-F1 (5′-CGGCTCCTATCCGTCCGG-3′) and *phoC*-A-R1 (5′-CAACATCGCTTTGCCAGTG-3′), whereas *phoD* fragments were amplified with ALPS-F730 (5′-CAGTGGGACGACCACGAGGT-3′) and ALPS-R1101 (5′-GAGGCCGATCGGCATGTCG-3′) [26,27,30,59]. PCR conditions consisted of an initial denaturation at 95 °C for 3 min; 35 cycles at 95 °C for 30 s, 53 °C for 30 s, and 72 °C for 45 s; and a final extension at 72 °C for 10 min. Amplicons were sequenced on an Illumina MiSeq platform by Majorbio (Shanghai, China). Paired-end reads were assembled with FLASH v1.2.11, followed by quality filtering in QIIME v1.9.1 [60]. High-quality sequences were grouped into operational taxonomic units (OTUs) at 97% sequence similarity using UPARSE v11.0 [25,61]. Before calculating the Simpson dominance index in QIIME v1.9.1, all samples were rarefied to the minimum sequencing depth. Specifically, the *phoC* dataset was rarefied to 25,007 sequences per sample, and the *phoD* dataset was rarefied to 18,949 sequences per sample. The raw reads, high-quality reads and rarefaction curves are presented in the Supplementary Materials (Table S1, Figure S1). The sequence data were deposited in NCBI (PRJNA1516858, PRJNA1517180).

### 2.6. Statistical Analysis

Differences in measured variables between the CK and Y20 treatments were evaluated using two-sided independent-samples *t*-tests in SPSS v26.0 (SPSS, Chicago, IL, USA), with four independent composite soil samples per treatment. The independence of observations was ensured because samples were collected from four separate sites for each treatment. Data distributions were assessed using the Shapiro–Wilk test, and homogeneity of variances was evaluated using Levene’s test. Treatment-related variation in *phoC*- and *phoD*-harboring bacterial communities was visualized by principal coordinate analysis (PCoA) based on Bray–Curtis dissimilarities, and its statistical significance was assessed using Adonis. The analysis was performed with 999 unrestricted permutations, and pseudo-F, R^2^, and permutation-based *p*-values were recorded. Homogeneity of multivariate dispersion was evaluated using the betadisper function before interpreting the PERMANOVA results. For subsequent correlation analyses, the scores of the first PCoA axis were used as a univariate summary of communities-structure variation because PCoA1 explained the largest proportion of the total variation among the axes. The PCoA1 explained 56.38% and 37.2% of the variation in the *phoC*- and *phoD*-harboring communities, respectively. Spearman correlations were calculated to characterize the associations among organic P forms, phosphatase activities, and the two functional bacterial communities. Because multiple Spearman correlation analyses were performed with only eight independent samples, no adjustment for multiple comparisons was applied. Therefore, the resulting *p* values should be interpreted as exploratory rather than confirmatory.

## 3. Results

### 3.1. Soil P Pools

The contents of TP, OP and MBP were in the range of 425.78–825.45, 83.30–185.89 and 7.08–10.19 mg kg^−1^, respectively, with no significant difference between CK and Y20 treatments (Figure 1A–C, *p* > 0.05). The average AP content was 15.83 mg kg^−1^ in CK soils and 40.50 mg kg^−1^ in Y20 soils, representing a significant difference between the two land-use types (Figure 1D, *p* < 0.05).

### 3.2. Soil P Forms Determined by ^31^P-NMR

**Figure 2:**
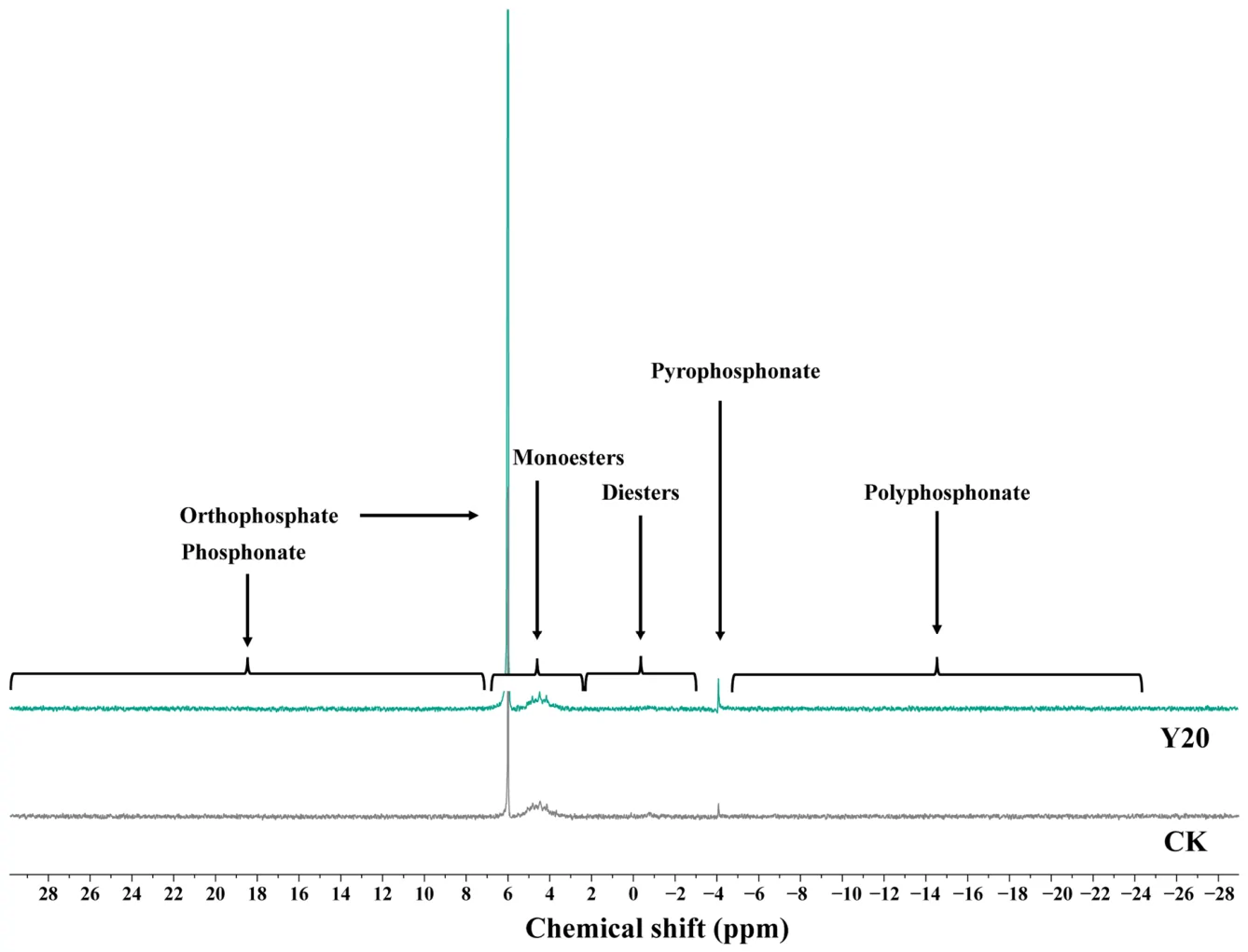
Liquid-state ^31^P NMR spectra of one representative sample from each of the uncultivated and grape orchard soils. CK: uncultivated soils; Y20: 20-year cultivated vineyard.

**Figure 3:**
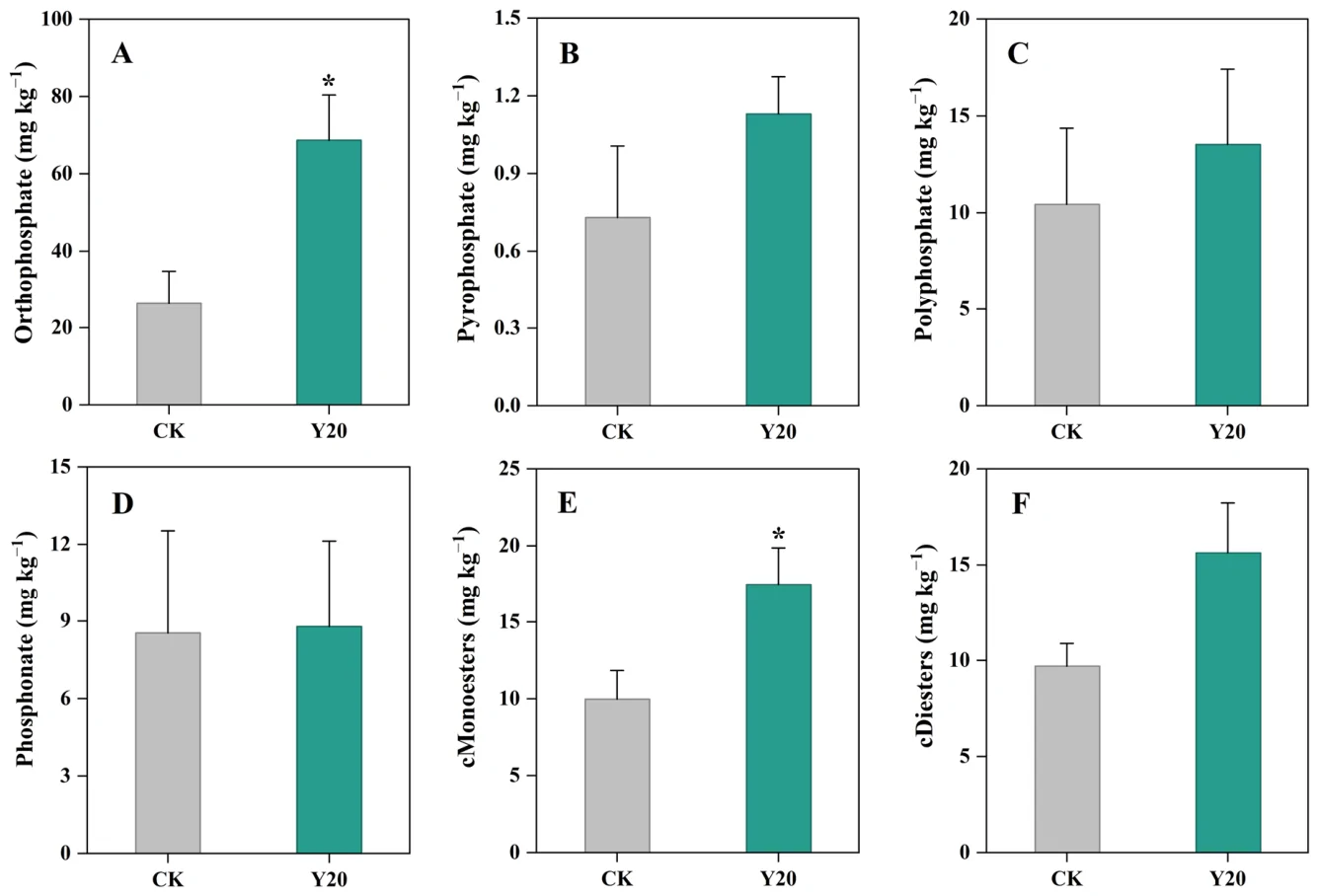
Mean concentrations of soil P forms determined by solution ^31^P-NMR spectroscopy in CK and Y20. Values are means and error bars represent standard errors (*n* = 4). (**A**) orthophosphate; (**B**) pyrophosphate; (**C**) polyphosphate; (**D**) phosphonate; (**E**) cMonoesters: corrected monoesters; (**F**) cDiesters: corrected diesters. CK: uncultivated soils; Y20: 20-year cultivated vineyard. * indicates significant differences between CK and Y20 (*p* < 0.05).

The soil inorganic P forms, determined by ^31^P-NMR, primarily consisted of orthophosphate, pyrophosphate and polyphosphate (Figure 2). Orthophosphate concentrations ranged from 15.34 to 97.45 mg kg^−1^ and were significantly higher in Y20 than in CK (Figure 3A, *p* < 0.05). The concentrations of pyrophosphate and polyphosphate varied from 0.31 to 1.54 and from 1.38 to 22.41 mg kg^−1^, respectively, with no significant difference between the two treatments (Figure 3B,C, *p* > 0.05).

The soil organic P forms, also determined by ^31^P-NMR, included phosphonate, monoester and diester (Figure 2). The CK and Y20 treatments exhibited phosphonate concentrations within the range of 0.72–19.99 mg kg^−1^, and there was no significant difference between them (Figure 3D, *p* > 0.05). The corrected monoester (cMonoester) concentrations ranged from 6.90 to 24.49 mg kg^−1^, accounting for 30% to 55% of organic P forms (Figure 3E). Compared with CK treatment, the cMonoester concentrations were significantly higher under Y20 treatment (Figure 3E, *p* < 0.05). The corrected diester (cDiester) concentrations varied from 6.90 to 23.27 mg kg^−1^, representing 27% to 45% of organic P forms, with no significant difference between the two treatments (Figure 3F, *p* > 0.05).

**Figure 4:**
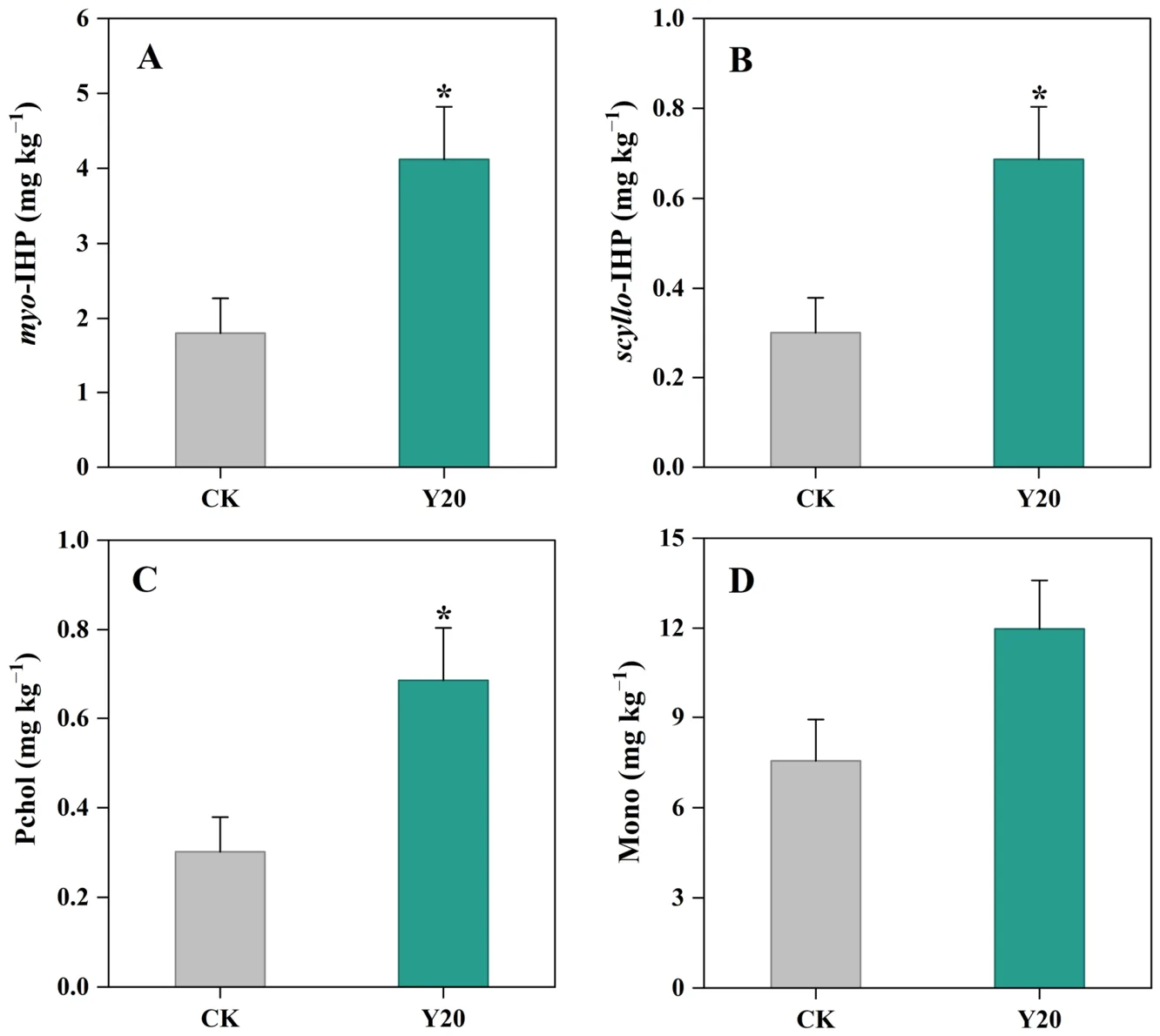
Mean concentrations of soil monoesters region in CK and Y20. Values are means and error bars represent standard errors (*n* = 4). (**A**) *myo*-IHP: *myo*-inositol hexakisphosphate; (**B**) *scyllo*-IHP: *scyllo*-inositol hexakisphosphate; (**C**) Pchol: choline phosphate; (**D**) Mono: unspecified monoesters. CK: uncultivated soils; Y20: 20-year cultivated vineyard. * indicates significant differences between CK and Y20 (*p* < 0.05).

The monoester area included *myo*-IHP, *scyllo*-IHP, Pchol and unspecified monoesters (Mono) (Figure 4). The average concentrations of *myo*-IHP, *scyllo*-IHP and Pchol in the Y20 treatment were 4.12, 0.69 and 0.69 mg kg^−1^, respectively, significantly higher than those observed in the CK treatment (Figure 4A–C, *p* < 0.05). The Mono concentrations ranged from 5.10 to 16.69 mg kg^−1^, and there was no significant difference between CK and Y20 treatments (Figure 4D, *p* > 0.05).

### 3.3. Soil Phosphatase Activities and Communities Diversity of phoC and phoD Genes

**Figure 5:**
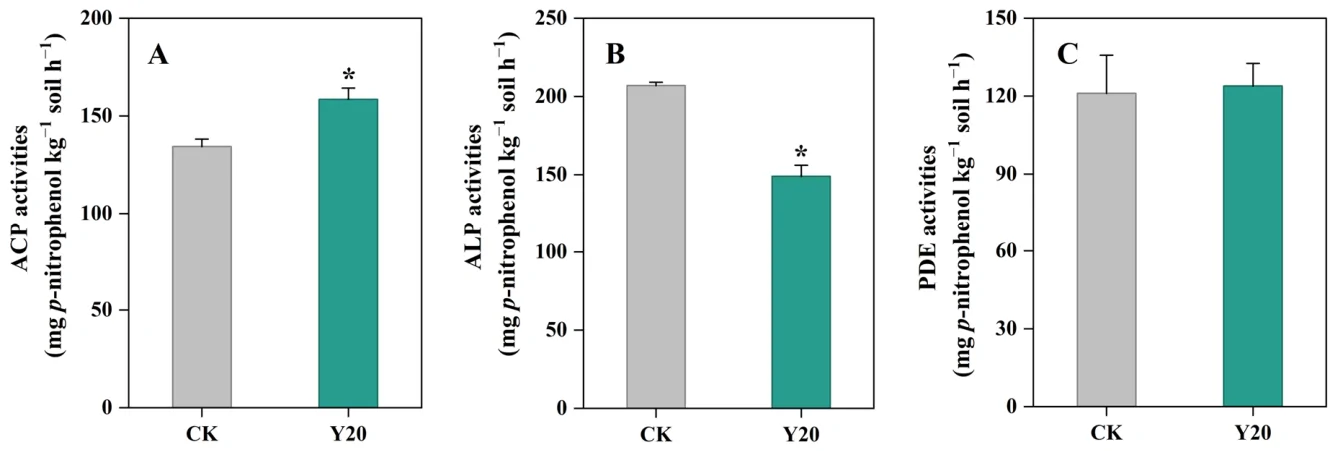
Changes in soil phosphatase activities under CK and Y20. Values are means and error bars represent standard errors (*n* = 4). (**A**) ACP: acid phosphomonoesterase; (**B**) ALP: alkaline phosphomonoesterase; (**C**) PDE: phosphodiesterase. CK: uncultivated soils; Y20: 20-year cultivated vineyard. * indicates significant differences between CK and Y20 (*p* < 0.05).

**Figure 6:**
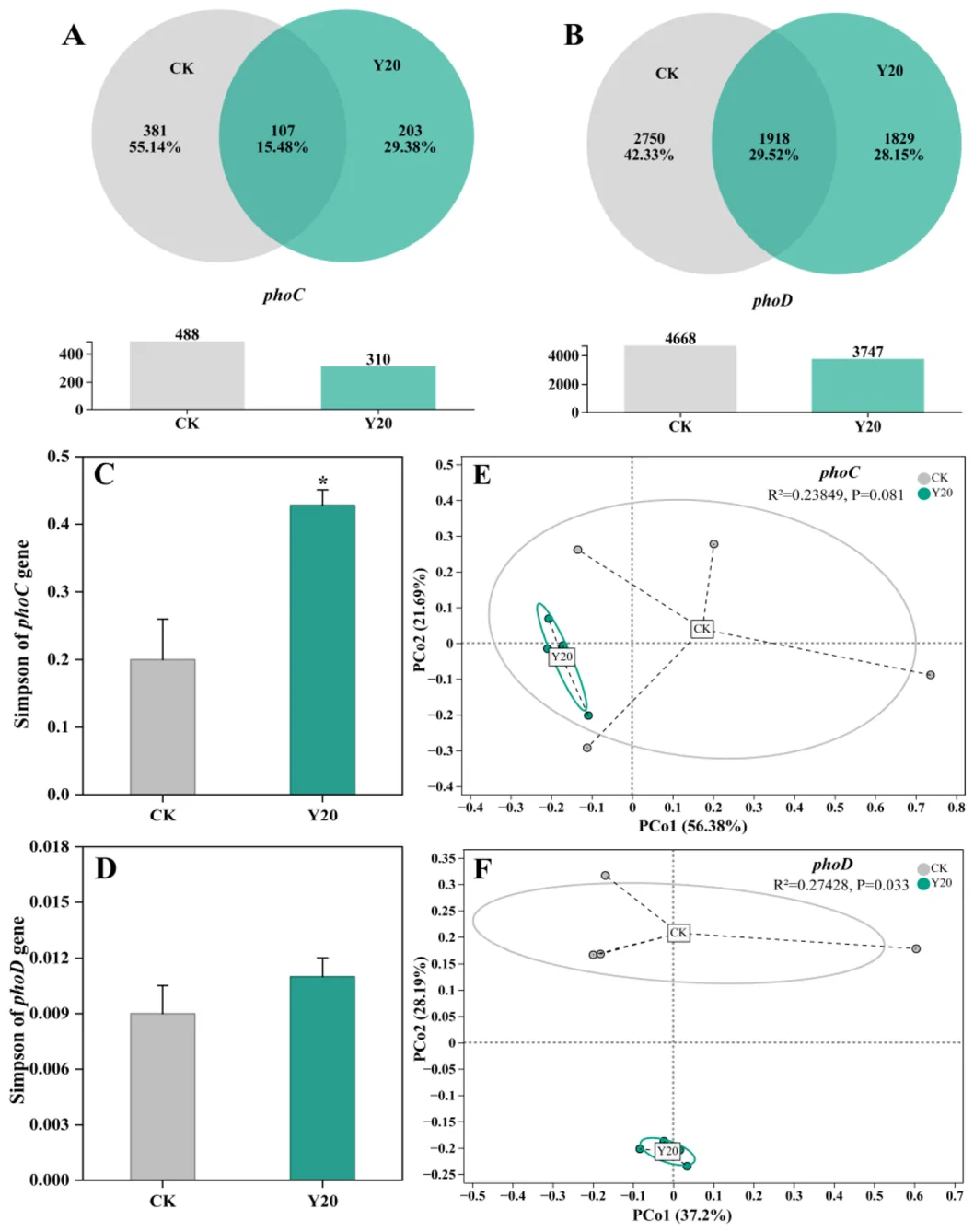
The shared and unique OTUs, α- and β-diversity of *phoC*- (**A**,**C**,**E**) and *phoD*- (**B**,**D**,**F**) harboring bacterial communities in CK and Y20. α-diversity was assessed via the Simpson dominance index. To compare β-diversity between treatments, principal coordinate analysis (PCoA) was performed using Bray–Curtis distances, with 95% confidence ellipses plotted for each group. Group dissimilarities were statistically evaluated using Adonis testing. Values are means and error bars represent standard errors (*n* = 4). CK: uncultivated soils; Y20: 20-year cultivated vineyard. * indicates significant differences between CK and Y20 (*p* < 0.05).

Compared with CK, soil ACP and ALP activities were significantly higher and lower under Y20 treatment, respectively (Figure 5A,B, *p* < 0.05). Soil PDE activity showed no significant difference between the two treatments (Figure 5C, *p* > 0.05). Remarkable differences in the OTU counts were observed between *phoC*-harboring bacteria (691 OTUs) and *phoD*-harboring bacteria (6497 OTUs) (Figure 6A,B). Only 15.48% of *phoC*-harboring OTUs were shared across the two treatments (Figure 6A), compared to 29.52% for *phoD*-harboring bacteria (Figure 6B). Compared with CK soils, Y20 soils contained 25.76% and 14.18% fewer *phoC*- and *phoD*-harboring OTUs, respectively (Figure 6A,B).

The Simpson dominance index was used to estimate the α-diversity of *phoC*- and *phoD*-harboring bacterial communities. The Simpson dominance index of the *phoC*-harboring bacterial communities differed significantly between the two land-use types and was higher in Y20 than in CK soils (Figure 6C, *p* < 0.05). However, the Simpson dominance index of *phoD*-harboring bacteria did not differ significantly between CK and Y20 treatments (Figure 6D, *p* > 0.05). Moreover, PCoA and Adonis analyses suggested that the *phoC* communities were similar across the treatments (Figure 6E, *p* > 0.05), while *phoD* communities were significantly different between the CK and Y20 treatments (Figure 6F, *p* < 0.05).

### 3.4. phoC- and phoD-Harboring Bacterial Community Composition

**Figure 7:**
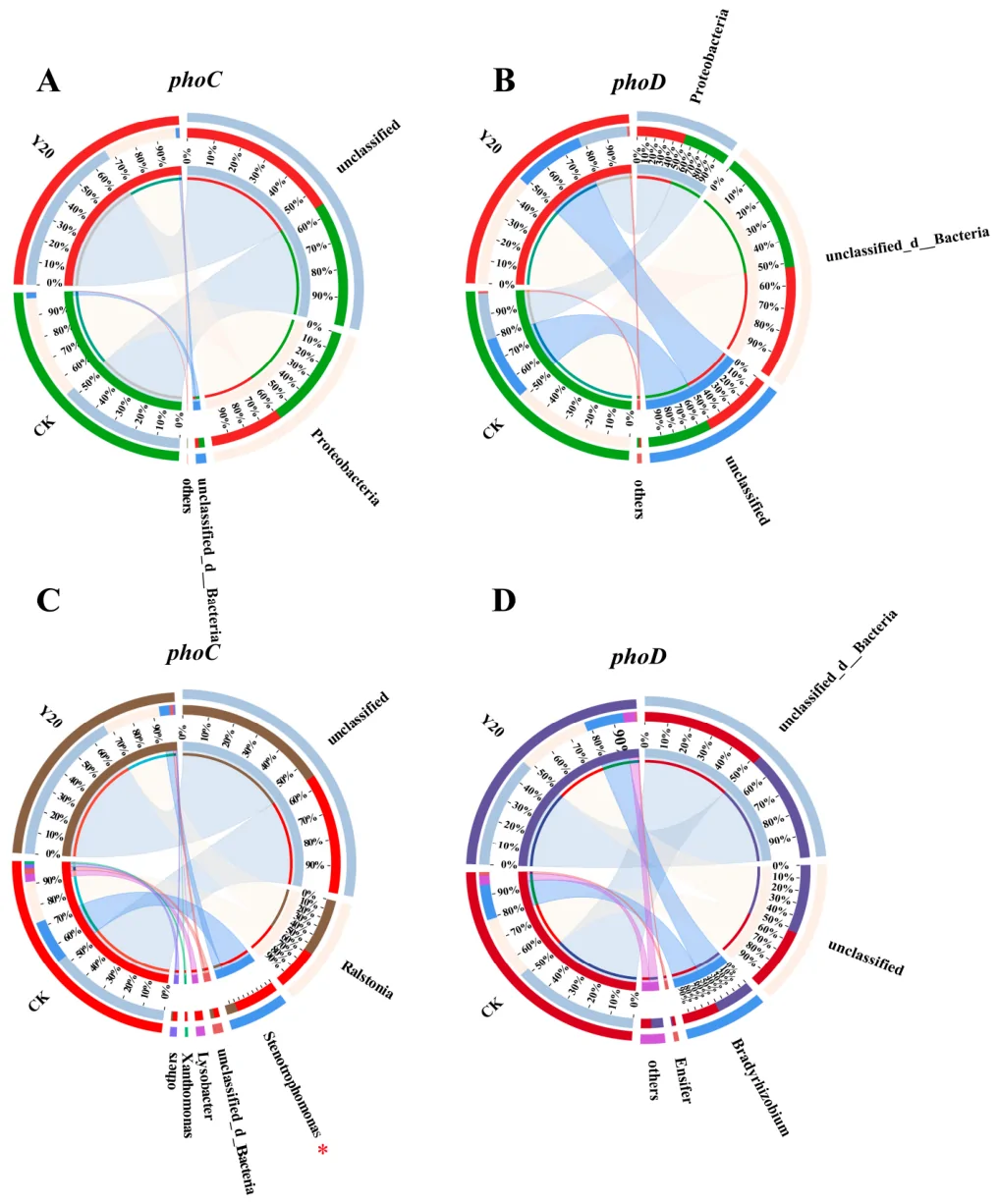
The main phyla and genera containing *phoC* (**A**,**C**) and *phoD* (**B**,**D**) in CK and Y20. CK: uncultivated soils; Y20: 20-year cultivated vineyard. * indicates significant differences between CK and Y20 (*p* < 0.05).

At the phylum level, *Proteobacteria* dominated both *phoC*- and *phoD*-harboring bacterial communities across all samples, and similar relative abundance was observed between CK and Y20 treatments (Figure 7A,B, *p* > 0.05). For the *phoC*-harboring community composition, *Ralstonia* and *Stenotrophomonas* were the dominant genera (Figure 7C). *Bradyrhizobium* and *Ensifer* were the dominant genera of the *phoD*-harboring communities (Figure 7D). The relative abundance of *Stenotrophomonas* significantly decreased in Y20 compared to CK (Figure 7C, *p* < 0.05), and the abundances of other genera were similar across the treatments (Figure 7C,D, *p* > 0.05).

### 3.5. Responses of Organic P Forms to Phosphatase Activities and phoC- and phoD-Harboring Bacterial Communities

**Figure 8:**
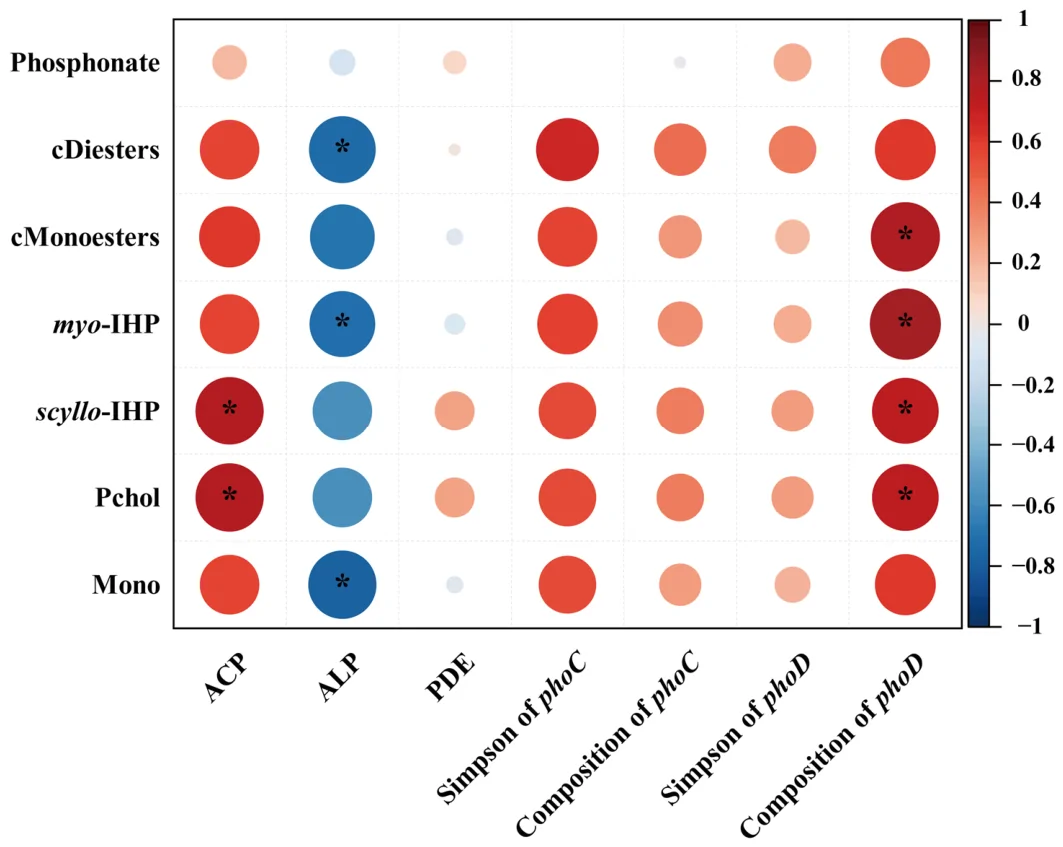
Correlation heatmap of soil organic P forms, phosphatase activities and *phoC-* and *phoD*-harboring bacterial communities in uncultivated and grape orchard soils (*n* = 8). Colors indicated the direction of the correlation. cDiesters: corrected diesters; cMonoesters: corrected monoesters; *myo*-IHP: *myo*-inositol hexakisphosphate; *scyllo*-IHP: *scyllo*-inositol hexakisphosphate; Pchol: Choline phosphate; Mono: unspecified monoesters; ACP: acid phosphomonoesterase; ALP: alkaline phosphomonoesterase; PDE: phosphodiesterase. * Correlation is significant at the 0.05 level.

Spearman’s correlation revealed the relationships between organic P forms determined by solution ^31^P NMR spectroscopy, phosphatase activities and *phoC*- and *phoD*-harboring bacterial communities (Figure 8). The results showed that soil ACP activity was significantly and positively related to the contents of *scyllo*-IHP and Pchol, while soil ALP activity was significantly and negatively correlated with cDiesters, *myo*-IHP and Mono (Figure 8, *p* < 0.05). With respect to *phoD*-harboring bacterial community composition, significant and positive correlations with cMonoesters, *myo*-IHP, *scyllo*-IHP and Pchol were also found (Figure 8, *p* < 0.05).

## 4. Discussion

### 4.1. Effects of Long-Term Vineyard Cultivation on Soil P Pools and Availability

**Figure A2:**
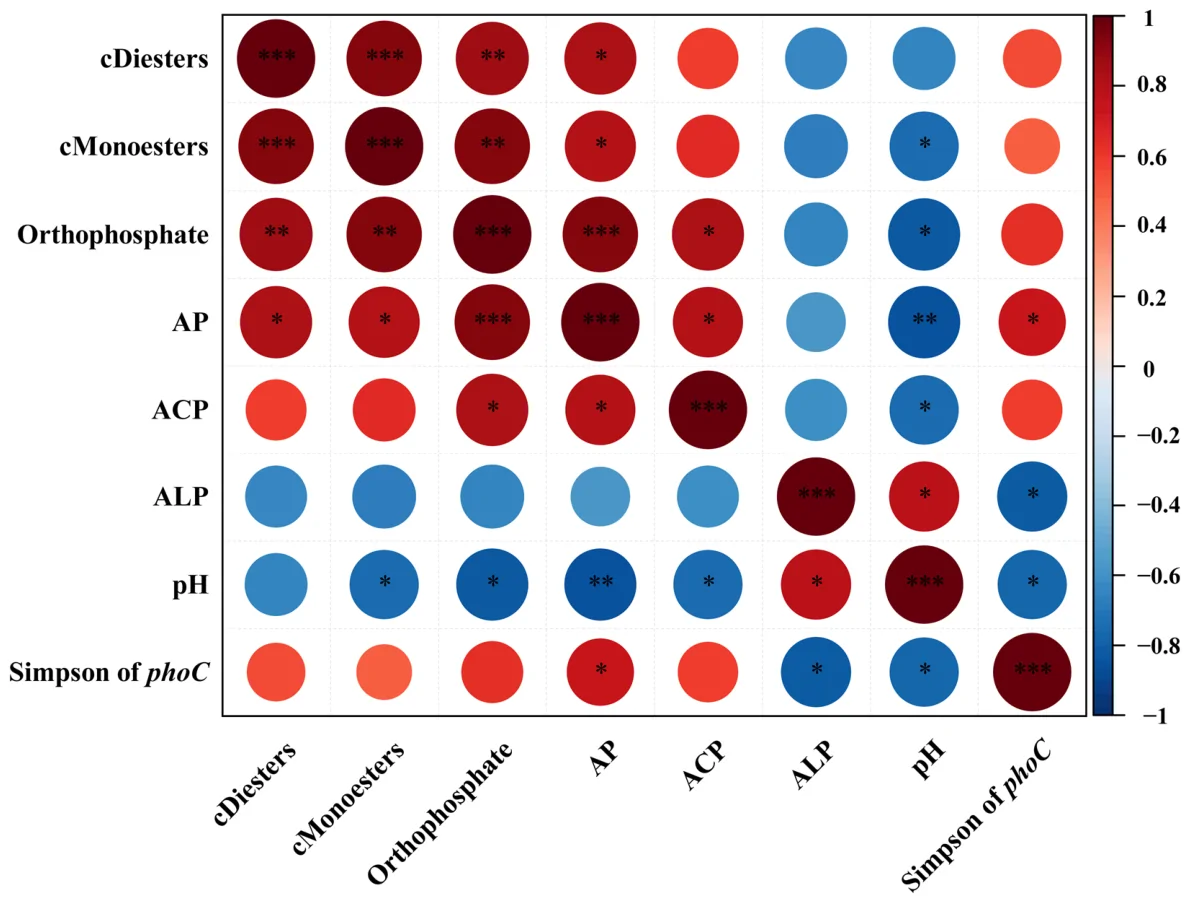
Heatmap of correlations between soil properties in uncultivated soils and grape orchard soils (*n* = 8). ACP: acid phosphomonoesterase; ALP: alkaline phosphomonoesterase; cMonoesters: corrected monoesters; cDiesters: corrected diesters; AP: available P. Values of pH were obtained from Fang et al. [1]. Red and blue indicate positive and negative correlations, respectively, with darker colors representing stronger correlations. * Correlation is significant at the 0.05 level. ** Correlation is significant at the 0.01 level. *** Correlation is significant at the 0.001 level.

Soil AP was markedly higher in the 20-year-old vineyard soils than in the adjacent uncultivated soils, whereas TP, OP, and MBP did not differ significantly between the two land-use types. This contrasting pattern suggests that long-term vineyard management was associated with changes in the internal distribution and bioavailability of soil P rather than with an expansion of the bulk soil P pool. Several mechanisms may help explain the higher AP concentration observed in the long-term vineyard soils. First, regular P fertilization during the 20-year vineyard management period may have contributed to the higher inorganic P availability observed in the vineyard soils, thereby leading to the observed accumulation of AP in the Y20 treatment. Second, the elevated soil AP concentration in Y20 largely stemmed from the lower pH of this treatment. Soil acidity reduces phosphate adsorption by weakening cation-oxygen bonds, which facilitates cation release and enhances P availability [62,63]. Third, AP was positively associated with orthophosphate, cMonoesters, and cDiesters (Figure A2). These relationships are consistent with the possibility that OP mineralization contributed to the AP pool; however, because P transformation rates and fluxes were not measured, OP mineralization cannot be identified as the direct source of the observed AP increase. Thus, the increase in AP should be interpreted as an observed change in P availability that may reflect the combined effects of fertilizer inputs, soil acidification, sorption–desorption processes, and OP turnover.

### 4.2. Changes in Soil P Speciation Following Long-Term Vineyard Cultivation

The solution ^31^P-NMR results indicated that soil P speciation differed between the long-term vineyard and uncultivated soils. As expected, orthophosphate was the predominant form of P in vineyard soil, which was in agreement with the findings from previous studies [13,22,64]. The higher orthophosphate concentration in the long-term vineyard soils was consistent with greater representation of labile inorganic P in these soils, although the underlying processes cannot be established conclusively from this space-for-time comparison. This phenomenon likely arose from the substantial input of inorganic P fertilizer, while lower soil pH facilitated the release of adsorbed P through desorption processes [62,63,65,66]. Moreover, cMonoester and cDiester contents were positively associated with orthophosphate content. This pattern is compatible with the hypothesis that phosphatase-mediated hydrolysis may contribute to orthophosphate formation, but it cannot distinguish enzymatic transformation from shared responses to fertilizer inputs, soil pH, mineral interactions, or other covarying environmental factors.

Because degradation can occur during analysis, certain monoesters may derive from diesters (α-Glyc, β-Glyc, mononucleotides, and half of the Monoester 2 region) [22,23,58]. Accordingly, the cMonoester concentration is equal to the uncorrected monoester total minus the degradation, while the cDiester concentration is equal to the uncorrected diester total plus the degradation. A particularly important finding was the significant increase in cMonoesters under long-term vineyard cultivation, while cDiesters showed no significant difference between CK and Y20. Under soil conditions conducive to decomposition, diesters typically undergo rapid mineralization, whereas monoesters tend to bind tightly to soil particles, thereby limiting their degradation [14,57]. Hence, the enrichment of cMonoesters under Y20 indicates that long-term vineyard management was associated with the accumulation of relatively stable organic P pools, despite elevated levels of soil AP and orthophosphate. The concurrent increase in P availability and cMonoester storage may reflect the coexistence of several processes, including continued organic P inputs or transformation, differential stabilization of monoesters, and possible enzymatic release of orthophosphate. The observed accumulation of cMonoesters may indicate that their inputs and/or physicochemical stabilization outweighed their removal or mineralization under the conditions prevailing in Y20 soils. Direct measurements of cMonoester production, stabilization, and mineralization rates would be needed to test this interpretation. This result aligned with Wu et al. [23], who reported that the no-tillage with 33% residue application treatment exhibited the highest soil AP concentration and significantly higher cMonoester concentrations than other treatments. Such an increase can be attributed to the increased litter inputs in perennial vineyard, as monoesters in the terrestrial environment are primarily synthesized in plants [67]. A second possible explanation is the lower soil pH observed under the Y20 treatment, in line with reports that soil metal oxides help maintain monoester concentrations in acidic soils [13,21]. The high charge density of monoesters favors their association with Fe/Al oxides and organic matter through precipitation or adsorption, thereby rendering them less accessible to microbial utilization and enzymatic breakdown [20,68]. In addition, PDE-mediated hydrolysis represents one possible pathway through which diester-derived products could contribute to the monoester pool [69]. The positive association between cDiesters and cMonoesters is compatible with this possibility, but does not demonstrate that PDE-mediated hydrolysis caused the observed cMonoester accumulation.

Previous research has demonstrated IHP as the most abundant identifiable organic P compound in the plough layer of cultivated soils [70]. Likewise, *myo*-IHP was the predominant monoester form in the present study, with significantly elevated concentrations in Y20. Soil *myo*-IHP is a major form of inositol phosphate derived largely from plant residues, seeds and microbial products, and it can be strongly adsorbed by soil minerals in acidic environments, especially Fe/Al oxides, making it relatively resistant to degradation [67,71,72]. Accordingly, the observed increase in Y20 could be ascribed to the significantly lower soil pH, which facilitated *myo*-IHP adsorption, and to the accumulated litter input, which enhanced its plant-derived sources. Soil *scyllo*-IHP followed a similar trend after the conversion of uncultivated land to long-term vineyard. As a stereoisomer that can be formed by epimerization of *myo*-IHP, *scyllo*-IHP may also persist in soil through mineral adsorption and precipitation with metal ions [71,73].

### 4.3. Responses of Soil Phosphatase Activities to Long-Term Vineyard Cultivation

The two phosphomonoesterases showed contrasting patterns in the long-term vineyard soils: ACP activity was higher, whereas ALP activity was lower than in the CK soils. These divergent patterns were probably associated with the shift in soil pH induced by vineyard establishment, because pH can alter enzyme conformation, substrate affinity, inhibition processes, and overall catalytic efficiency [37]. Soil phosphatase activity is also influenced by microbial functioning and the relative contribution of plant- and microbial-derived enzymes. In general, ACP is more prevalent under acidic conditions, whereas ALP tends to be favored in neutral to alkaline soils [38,39]. Consistent with this ecological pattern, ACP activity was significantly and negatively correlated with soil pH, while ALP activity showed a significant positive correlation with pH in the present study (Figure A2). Although some agricultural soils exhibit enhanced ACP activity following acidification [40], other studies have reported inhibitory effects of declining pH on ACP activity and P-cycling enzymes [41,42]. These contrasting findings indicate that the effect of acidification on phosphatase activity is context-dependent and may be regulated by multiple interacting factors. In our study, the lower pH in Y20 may have provided more favorable conditions for ACP functioning, potentially in combination with the extensive grapevine root system and its associated rhizosphere processes. Phosphatases can be produced by both plants and microorganisms; however, ACP is generally considered to originate from plant roots and microbial communities, whereas ALP is predominantly associated with microorganisms [39]. The simultaneous increase in ACP activity and decline in *phoC* gene diversity in Y20 may be consistent with a possible contribution from plant roots. However, this interpretation remains tentative because plant- and microbial-derived phosphatase activities were not separated, and *phoC* abundance or expression was not measured. Therefore, the present data do not allow us to determine whether the increased ACP activity was primarily derived from plants or microorganisms. The observed pattern may reflect changes in the relative contributions of plant roots, microorganisms, and extracellular enzyme stabilization in the soil. Other possibilities include functional redundancy among *phoC*-harboring bacteria, increased activity per taxon, altered enzyme persistence, and changes in soil pH or substrate availability. Direct measurements of *phoC* abundance and transcription, together with methods that distinguish plant-derived from microbial phosphatase activity, will be required to resolve the origin of the increased ACP activity. In contrast, the lower ALP activity in Y20 may be related to the higher AP concentration observed in these soils and concurrent changes in the composition of the *phoD*-harboring bacterial communities [74]. This interpretation is consistent with the negative feedback relationship commonly reported between ALP activity and inorganic P availability, whereby elevated P availability reduces the microbial investment in ALP production [8,34]. Finally, PDE activity remained statistically unchanged between treatments, indicating that the hydrolysis potential of diester P was relatively stable following long-term vineyard cultivation. This result agrees with the lack of significant differences in soil cDiester concentrations.

### 4.4. Differential Responses of phoC- and phoD-Harboring Bacterial Communities to Long-Term Vineyard Cultivation

The *phoC*- and *phoD*-carrying bacterial assemblages exhibited different patterns in soils associated with long-term vineyard management. Across all samples, substantially fewer OTUs were detected for the *phoC* assemblage than for its *phoD* counterpart, indicating a narrower observed taxonomic representation of the *phoC*-associated assemblage. However, OTU richness alone cannot be used to infer differences in absolute gene abundance or functional redundancy. A similar disparity was reported in soils under long-term citrus cultivation, where the number of *phoC* OTUs was considerably lower than that of *phoD* OTUs [8]. The Y20 soils contained fewer OTUs belonging to both functional groups than the CK soils, with a greater reduction in observed OTU richness for the *phoC*-associated assemblage. Consistent with this pattern, Y20 exhibited significantly lower α-diversity of the *phoC* assemblage, as indicated by a higher Simpson dominance index, whereas no comparable change was detected for *phoD*. This decline may be related to the lower pH and higher AP concentrations observed in the Y20 soils, although these relationships should not be interpreted as definitive causal effects. Correlation analysis further showed that the Simpson dominance index of *phoC*-harboring communities exhibited significantly negative and positive relationships with soil pH and AP, respectively (Figure A2).

**Figure A3:**
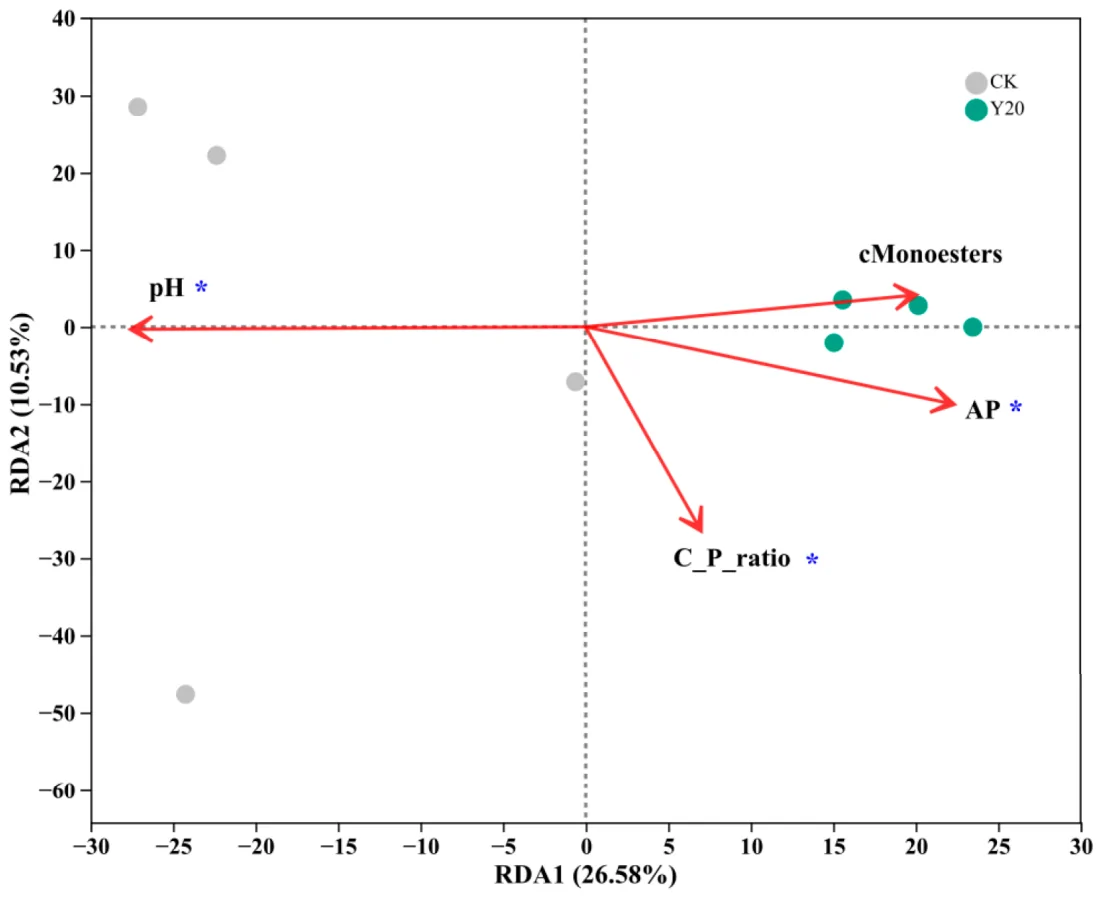
Redundancy analysis (RDA) assessing the relationship between *phoD* gene community structure and soil properties. AP: available P; cMonoesters: corrected monoesters; C_P_ratio: ratio between soil organic carbon (SOC) and soil total P. Values of pH and SOC were obtained from Fang et al. [1]. CK: uncultivated soils; Y20: grape orchards cultivated for 20 years. * indicates statistically significant soil properties at *p* < 0.05.

Despite the greater decline in *phoC* α-diversity, the Y20-CK comparison revealed a more pronounced shift in the overall composition of the *phoD*-carrying bacterial assemblage. Specifically, the *phoD* communities differed significantly between CK and Y20, while the response of the *phoC* communities was comparatively limited. This contrast supports the view that the responses of P-cycling functional groups to agricultural management are taxon-dependent [25]. Several mechanisms may explain their differential sensitivity. First, plant roots can contribute substantially to ACP production, potentially buffering microbial *phoC*-mediated functions against environmental disturbances [26]. Second, bacteria carrying *phoC* and *phoD* may occupy distinct ecological niches and differ in their resource requirements [25]. Many *phoC*-carrying genera exhibit oligotrophic traits and can persist under nutrient-limited conditions, which may confer greater resistance to environmental variation [44,45]. By contrast, the composition of the *phoD* assemblage appears to be more responsive to edaphic conditions. Previous studies have identified soil pH, AP, C:P stoichiometry, and organic P substrates as major determinants of *phoD*-carrying bacterial communities [10,27,43,46,47]. The significant relationships observed between these variables and the community composition of *phoD* in the present study provide further support for this interpretation (Figure A3).

At the phylum level, *Proteobacteria* predominated in both functional assemblages, highlighting their potentially important contribution to organic P turnover in uncultivated and vineyard soils [75,76,77]. At the genus level, however, *Stenotrophomonas* was the only member of the *phoC* assemblage whose relative abundance declined significantly in Y20. Members of this genus are known for their plant growth-promoting properties and their capacity to mobilize soil P [78,79]. In acidic soils, *Stenotrophomonas* has been identified as an important ACP producer and a potentially influential taxon within the *phoC*-carrying communities [25]. Some strains can utilize relatively recalcitrant organic P compounds, including phosphonates and phytate [80,81]. In addition, Al-tolerant members of this genus may mitigate metal toxicity under acidic conditions through organic acid secretion and siderophore production [82]. Their reported ability to solubilize inorganic P further suggests that *Stenotrophomonas* may participate in multiple pathways of soil P mobilization, encompassing both organic P mineralization and inorganic P solubilization [83,84]. Therefore, the lower relative abundance of *Stenotrophomonas* in Y20 may alter the relative contribution of *phoC*-carrying bacteria to P transformation, although the functional consequences of this taxonomic shift require further verification.

A potential limitation of this study is that the long-term application history of Cu-based fungicides and the resulting soil Cu concentrations were not available. Vineyard soils may accumulate Cu after repeated fungicide application, and Cu enrichment could affect bacterial diversity, community composition, and phosphatase-related microorganisms. Therefore, the observed differences in the *phoC*- and *phoD*-harboring bacterial communities cannot be attributed exclusively to soil pH, AP, C:P, or other measured soil properties. Instead, these variables should be regarded as potential correlates of the microbial responses under long-term vineyard cultivation. Future studies should combine soil Cu measurements, detailed pesticide-use records, and matched baseline or chronosequence sampling to distinguish the effects of vineyard cultivation from those of Cu accumulation.

### 4.5. Associations Among Soil Organic P Forms, Phosphatase Activities, and phoC- and phoD-Harboring Bacterial Communities

Correlation analyses further elucidated the relationships among organic P speciation, phosphatase activities, and functional bacterial communities. Soil ACP activity was positively associated with organic P pools, particularly *scyllo*-IHP and Pchol, consistent with previous findings [23,64]. This association is compatible with a substrate-related response, although substrate induction was not directly demonstrated. Long-term vineyard cultivation resulted in lower soil pH and increased litter inputs, conditions that may have promoted the accumulation of *scyllo*-IHP and Pchol [67,71,72]. As these phosphomonoesters are potential substrates for ACP, their enrichment may stimulate ACP production by plant roots and soil microorganisms [39]. In contrast, ALP activity was negatively associated with organic P pools. This inverse relationship is biologically plausible because ALP catalyzes the hydrolysis of phosphomonoesters to release orthophosphate; therefore, the reduced ALP activity observed under Y20 may constrain phosphomonoester turnover and consequently favor their accumulation [39,59]. Comparable patterns have also been documented in other studies [13,85]. Moreover, this relationship may reflect the concurrent regulation of ALP activity and organic P persistence by soil pH and available P (AP). Specifically, acidic conditions and high AP availability can suppress ALP production, thereby reducing ALP-mediated hydrolysis and promoting the retention of organic P compounds [8,34,38,39]. The composition of the *phoD*-harboring bacterial communities was also positively related to phosphomonoester pools, supporting the important role of these bacteria in soil P transformations and their sensitivity to soil P status [59,77,86,87]. Long-term vineyard cultivation was associated with shifts in the composition of *phoD*-associated bacterial communities, which may be related to changes in key edaphic variables, including soil pH, AP concentration, and the C:P ratio (Figure A3). Nevertheless, the shift in the composition of the *phoD*-harboring bacterial communities was not accompanied by a corresponding increase in ALP activity. This pattern may be consistent with a possible decoupling between genetic potential and realized enzymatic function; however, this interpretation remains a hypothesis because gene abundance and transcript data were not available. This discrepancy is expected because the presence of functional genes does not necessarily translate into enzyme production or activity, which can be further constrained by soil pH, P availability, microbial physiological status, and transcriptional and post-transcriptional regulation. Collectively, these results suggest that organic P dynamics were associated with ACP and ALP activities and with the composition of the *phoD*-associated bacterial assemblages. Nevertheless, the present data do not establish a direct functional role or changes in the absolute abundance of these bacterial groups.

This study has several limitations that should be considered when interpreting the observed patterns. First, the comparison involved four 20-year-old vineyards and four nearby uncultivated reference sites, with five soil cores combined into one composite sample at each site. Therefore, the site-level sample size was four independent replicates per land-use type. Second, because the study used a space-for-time comparison rather than a longitudinal experiment that followed the same sites before and after vineyard establishment, the observed differences cannot be interpreted as definitive causal effects of vineyard cultivation. Although the adjacent reference sites were selected to provide a comparison with the vineyards, unmeasured differences in initial soil properties, topography, parent material, vegetation history, and management practices may also have contributed to the observed patterns. Accordingly, the increases in AP, orthophosphate, cMonoesters, and ACP activity, as well as the changes in *phoC*- and *phoD*-harboring bacterial communities, should be interpreted as associations with long-term vineyard management rather than direct effects proven by experimental manipulation. Moreover, an important limitation concerns the interpretation of the correlation analyses. All Spearman correlations were calculated across the pooled dataset comprising four CK and four Y20 sites (*n* = 8). Because CK and Y20 soils were separated with respect to several soil and microbial variables, the resulting correlation coefficients may partly reflect between-treatment differences rather than covariation within treatments. In addition, the small sample size limits the precision of the correlation estimates and results in wide uncertainty around the corresponding coefficients. Although treatment-specific inspection of the scatterplots provides a visual assessment of this issue, the small number of sites per treatment (*n* = 4) precludes reliable within-treatment correlation testing (Figure S2). Therefore, the correlations presented here should be considered exploratory and hypothesis-generating rather than evidence of treatment-independent relationships or causal mechanisms. Future studies based on long-term monitored plots, chronosequences with more planting-age classes, or replicated field experiments are needed to disentangle the effects of vineyard age, fertilization, soil acidification, root activity, and other co-occurring management factors.

## 5. Conclusions

Compared with adjacent uncultivated soils, soils under 20-year vineyard management had significantly higher concentrations of AP, orthophosphate, and cMonoesters, particularly *myo*-IHP, *scyllo*-IHP, and Pchol, whereas TP, OP, and MBP did not differ significantly between the two land-use types. The long-term vineyard soils also exhibited higher ACP activity but lower ALP activity, while PDE activity remained unchanged. Furthermore, the *phoC*- and *phoD*-harboring bacterial communities exhibited distinct responses: the α-diversity of *phoC*-harboring communities was lower in Y20 soils, whereas the composition of *phoD*-harboring communities differed significantly between Y20 and CK soils. Soil organic P forms were closely associated with ACP and ALP activities and the composition of *phoD*-harboring communities. Overall, long-term vineyard management was associated with greater P availability and the accumulation of relatively stable monoester P, apparently through changes in the distribution of P among chemical forms rather than an expansion of the bulk soil P pool. However, because the study used a space-for-time comparison with four independent sites per land-use type, these results represent associations with long-term vineyard management and should not be interpreted as definitive causal effects of vineyard establishment. Long-term monitoring and replicated field experiments are needed to further resolve the mechanisms underlying P transformation in perennial vineyard soils.

## Data Availability

The original contributions presented in this study are included in the article/Supplementary Materials. Further inquiries can be directed to the corresponding authors.

## Supplementary Materials

The following supporting information can be downloaded at: https://www.mdpi.com/article/10.3390/microorganisms14091991/s1, Figure S1: Rarefaction curve analysis of the sequencing data for the *phoC* (A) and *phoD* (B) genes. CK: uncultivated soils; Y20: 20-year cultivated vineyard; Figure S2: Correlation heatmap of soil organic P forms, phosphatase activities and *phoC*- and *phoD*-harboring communities in uncultivated (A) and grape orchard soils (B) (*n* = 4). Colors indicated the direction of the correlation. CK: uncultivated soils; Y20: 20-year cultivated vineyard. cDiesters: corrected diesters; cMonoesters: corrected monoesters; *myo*-IHP: *myo*-inositol hexakisphosphate; *scyllo*-IHP: *scyllo*-inositol hexakisphosphate; Pchol: Choline phosphate; Mono: unspecified monoesters; ACP: acid phosphomonoesterase; ALP: alkaline phosphomonoesterase; PDE: phosphodiesterase; Table S1: Numbers of raw reads and high-quality reads obtained for the *phoC* and *phoD* genes.

## Abbreviations

The following abbreviations are used in this manuscript:

OP	Organic phosphorus
CK	Uncultivated reference sites
Y20	Soils from 20-year-old vineyards
AP	Available P
cMonoester	Corrected monoester
*myo*-IHP	*Myo*-inositol hexakisphosphate
ACP	Acid phosphomonoesterase
ALP	Alkaline phosphomonoesterase
P	Phosphorus
TP	Total P
NMR	Nuclear magnetic resonance
PDE	Phosphodiesterase
N	Nitrogen
MBP	Microbial biomass P
α-Glyc	α-glycerophosphate
β-Glyc	β-glycerophosphate
Pchol	Choline phosphate
OTUs	Operational taxonomic units
PCoA	Principal coordinate analysis
cDiesters	Corrected diester concentration

## Appendix A

**Table A1:** Chemical shifts of peaks detected in ^31^P-NMR spectra.

Category	P Form Compound Class	Chemical Shift/ppm
Inorganic P		
	Orthophosphate	6.00
	Pyrophosphate	−4.27 ± 0.02
	Polyphosphates	−5.00 ± 0.34–−25.00 ± 0.05
Organic P		
	Phosphonates	30.00 ± 0.07–7.00 ± 0.30
Orthophosphate monoesters		
	*myo*-inositol hexakisphosphate	5.65 ± 0.05, 4.74 ± 0.05, 4.36 ± 0.05, 4.23 ± 0.06
	*scyllo*-inositol hexakisphosphate	3.84 ± 0.03
	Choline phosphate	3.95 ± 0.03
	Monoester 1	6.90 ± 0.01–6.10 ± 0.01
	Monoester 2	5.90 ± 0.01–4.00 ± 0.02
	Monoester 3	3.90 ± 0.04–2.60 ± 0.01
Degradation compounds		
	α-glycerophosphate	4.95 ± 0.05
	β-glycerophosphate	4.64 ± 0.08
	Mononucleotides	4.53 ± 0.05, 4.51 ± 0.02, 4.47 ± 0.01, 4.43 ± 0.02
Orthophosphate diesters		
	DNA	−0.72 ± 0.05, −0.94 ± 0.04
	Diester 1	2.5 ± 0.35–−0.6 ± 0.04
	Diester 2	−1.2 ± 0.41–−3.7 ± 0.28
