# Supplementary material for: Long-Term Vineyard Cultivation Reshapes Soil Organic Phosphorus Speciation, Phosphatase Activities, and phoC- and phoD-Harboring Bacterial Communities
Source: Microorganisms. 2026 Sep 9;14(9):1991. doi: 10.3390/microorganisms14091991 (PMC13609722; doi:10.3390/microorganisms14091991)
Supplement: Supplementary file 1 [file microorganisms-14-01991-s001.zip › microorganisms-4507984-supplementary.pdf]

Table S1. Numbers of raw reads and high-quality reads obtained for the *phoC* and *phoD* genes

Gene	Sample	Raw reads	High-quality reads
phoC	CK1	47746	46164
	CK2	50570	48879
	CK3	57451	53579
	CK4	47982	46085
	Y20_1	60710	59760
	Y20_2	39238	35967
	Y20_3	40531	39826
	Y20_4	37433	36808
phoD	CK1	43918	29392
	CK2	46316	30149
	CK3	72610	46485
	CK4	38665	27557
	Y20_1	45062	27647
	Y20_2	44858	28206
	Y20_3	80002	41334
	Y20_4	35746	32045

CK: uncultivated soils; Y20: 20-year cultivated vineyard.

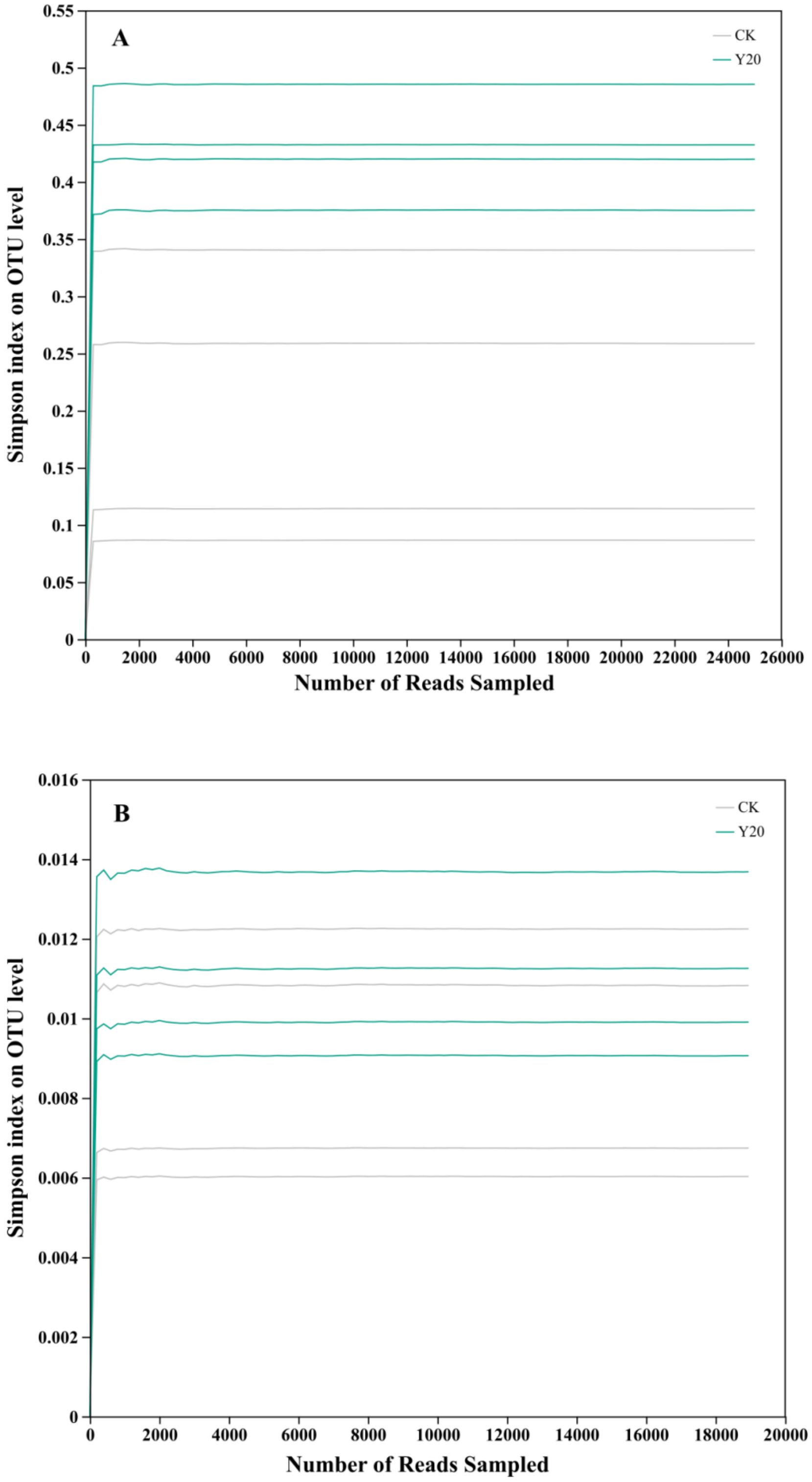

Figure S1. Rarefaction curve analysis of the sequencing data for the *phoC* (A) and *phoD* (B) genes. CK: uncultivated soils; Y20: 20-year cultivated vineyard.

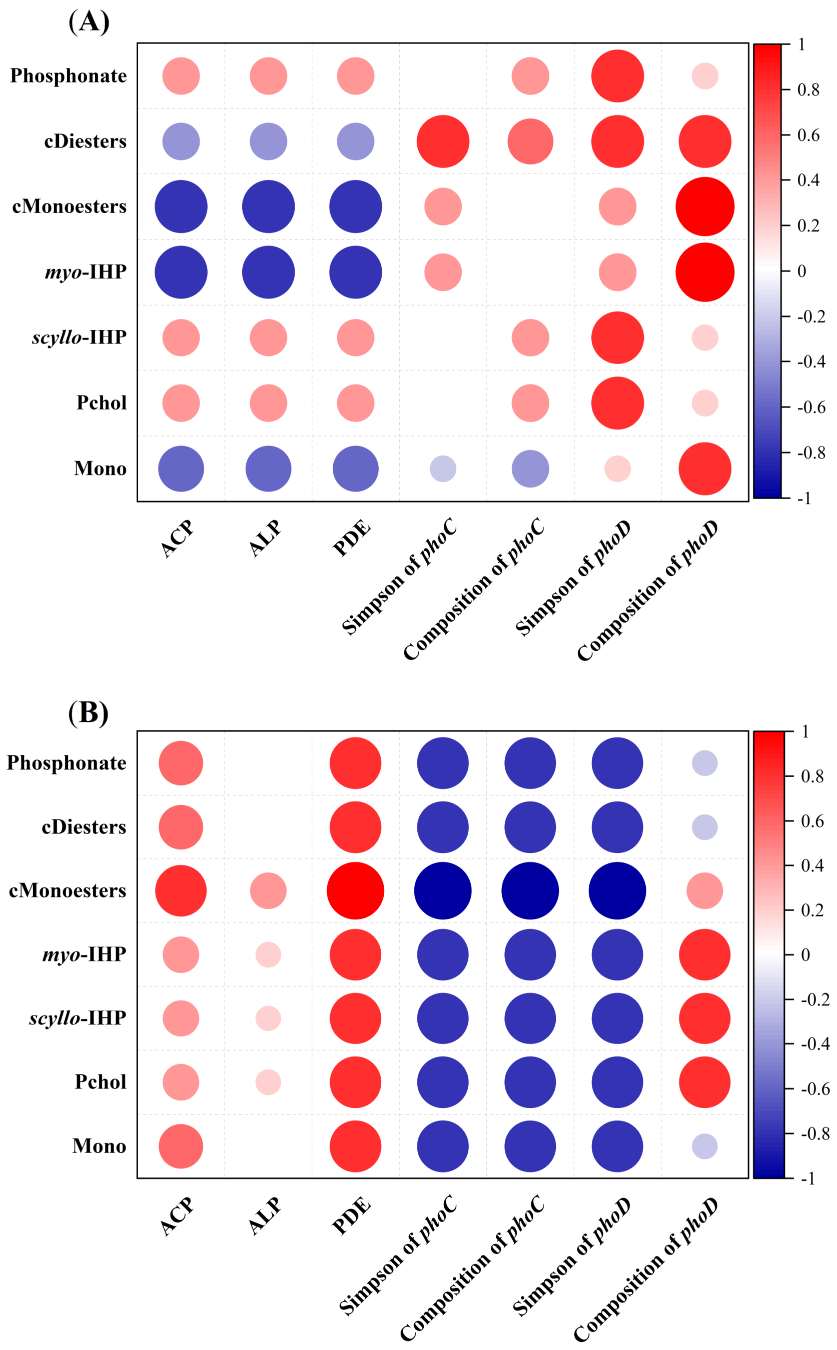

Figure S2. Correlation heatmap of soil organic P forms, phosphatase activities and *phoC*- and *phoD*-harboring communities in uncultivated (A) and grape orchard soils (B) (n=4). Colors indicated the direction of the correlation. CK: uncultivated soils; Y20: 20-year cultivated vineyard. cDiesters: corrected diesters; cMonoesters: corrected monoesters; *myo*-IHP: *myo*-inositol hexakisphosphate; *scyllo*-IHP: *scyllo*-inositol hexakisphosphate; Pchol: Choline phosphate; Mono: unspecified monoesters; ACP: acid phosphomonoesterase; ALP: alkaline phosphomonoesterase; PDE: phosphodiesterase.
